# Prediction of Short- to Long-Term Cyclic Deformation Behavior and Fatigue Life of Polymers

**DOI:** 10.3390/polym16121640

**Published:** 2024-06-10

**Authors:** Thierry Barriere, Stani Carbillet, Xavier Gabrion, Sami Holopainen

**Affiliations:** 1 CNRS, Institute FEMTO-ST, University of Franche-Comté, F-25000 Besançon, France; 2 SUPMICROTECH, CNRS, Institute FEMTO-ST, F-25000 Besançon, France; 3 Department of Civil Engineering, Tampere University, FI-33014 Tampere, Finland

**Keywords:** modeling and experimentation, ratcheting deformation, fatigue, plastic, model calibration

## Abstract

The prediction of mechanical behavior and fatigue life is of major importance for design and for replacing costly and time-consuming tests. The proposed approach for polymers is a combination of a fatigue model and a governing constitutive model, which is formulated using the Haward–Thackray viscoplastic model (1968) and is capable of capturing large deformations. The fatigue model integrates high- and low-cycle fatigue and is based on the concept of damage evolution and a moving endurance surface in the stress space, therefore memorizing the load history without requesting vague cycle-counting approaches. The proposed approach is applicable for materials in which the fatigue development is ductile, i.e., damage during the formation of microcracks controls most of the fatigue life (up to 90%). Moreover, damage evolution shows a certain asymptote at the ultimate of the low-cycle fatigue, a second asymptote at the ultimate of the high-cycle fatigue (which is near zero), and a curvature of how rapidly the transition between the asymptotes is reached. An interesting matter is that similar to metals, many polymers satisfy these constraints. Therefore, all the model parameters for fatigue can be given in terms of the Basquin and Coffin–Manson model parameters, i.e., satisfying well-defined parameters.

## 1. Introduction

The experimental determination of material properties is of major importance to the development of the most capable materials for use in demanding circumstances during their service lives. Development can be intensified by model predictions, with at least part of the costly and time-consuming tests able to be replaced by model predictions, i.e., model predictions can rapidly and systematically scan a vast number of material grades and loading situations. Examples of components that are manufactured from polymers and are subjected to cyclic fatigue loads range from uses in automotive and aeronautic equipment, healthcare instruments, marine structures, and sporting goods. The current value of the polymer market is huge—approximately USD 600 billion solely for the most common polymers—and it is further increasing [1]. Concurrently, fatigue failures of components have been evaluated to be the most important origin of immense financial losses [2,3,4]. Despite the huge importance of polymer engineering components, the volume of research for fatigue has concentrated mostly on metallic materials, while the fatigue characteristics of polymers are still under-researched, and much effort is required to enhance their fatigue resistance [5,6] and to develop accomplished simulation tools for failure assessment [7,8,9,10,11].

A capable simulation tool for fatigue must include a capable constitutive and fatigue damage model. Recent ambitious constitutive models for large deformations (without fatigue) are presented in [12,13,14,15,16,17,18]. For the investigation of both low- and high-cycle fatigue regimes in highly crystalline and semi-crystalline polymers, References [10,19] provided capable tools based on a hyperelastic–viscoplastic model and an elastic viscoplastic (parallel rheological network) model, respectively. However, when considering many polymers, especially amorphous (glassy) polymers, both the viscoelastic and plastic elements are required to accurately predict the long-term creep and recovery and, thus, the long-term cyclic and fatigue deformation behavior (shape of the loops and ratcheting) [20]. The models introduced in [7,21,22,23,24] include these elements, despite being demonstrated by ambiguous material parameter calibration.

An abundance of fatigue models rely on the fatigue-limit criteria wherein the fatigue strength or limit is determined by exploiting a set of identical cycles, and the models are equipped with cumulative damage theories and cycle-counting techniques [11,25,26]. However, it may be difficult to define a standard cycle from complex load spectra in practical applications. Another frequently applied concept is failure diagrams [27], e.g., Kitagawa-Takahashi, but their application to viscous polymers is difficult due to a certain controversy regarding the threshold and driving force for fatigue crack propagation [20]. Therefore, a continuum mechanics framework based on an incremental formalism is used in this work [28]. In contrast to cycle-counting methods, damage evolution and movement of the endurance surface are defined in terms of stress increments, not of stress cycles: the continuum mechanics framework is unified and consistent as it contains stress-based fatigue limits and the accumulation of damage for arbitrary stress histories (without a specific material-based constitutive theory).

**Figure 1:**
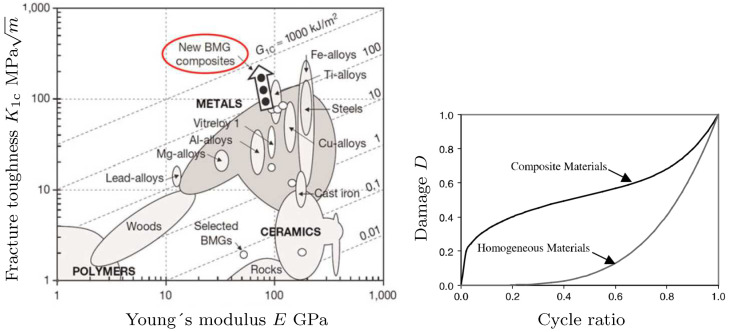
Ashby plot of the fracture toughness vs. Young’s modulus for a range of established engineering materials taken from [35] (**left**). Bulk metallic glasses (BMGs) and their composites show ultimate fracture toughness [35,38]. Sketched fatigue damage evolution of composites and homogeneous materials (incl. steels and polymers) [39] (**right**).

The present work focuses on modeling the cyclic deformation behavior and fatigue of solid polymers with well-defined parameter fitting based on principles used for metal fatigue, i.e., all of the model (material) parameters are defined using the celebrated Basquin-Coffin–Manson formulas, which are well defined in the fatigue literature [29,30]. This model calibration can be termed a physically grounded parameter calibration strategy for polymers. A similar idea has previously been applied to polymer foams [31], and [32] investigated the interaction between multi-axial ratcheting and fatigue in an ABS-polymer, showing that a slightly modified Basquin model can be applied. The motivation is that [1] many polymers satisfy the constraint that fatigue behavior is ductile, i.e., damage during the development of microcracks distinctively governs most of the fatigue life (even up to 90%) [23,33,34]. This property is correlated with a high proportion of fracture toughness, which is $K_{1}c/E∼1E-3$ m^1/2^ for polymers and, for instance, ∼0.5E−3 m^1/2^ for steels [35], cf. Figure 1(left). Accordingly, since polymers and metals are macroscopically homogeneous materials, they show constantly increasing accumulation of fatigue damage as demonstrated in Figure 1(right). Ref. [2] Many polymers also satisfy the prerequisite for the existence of an endurance limit and fatigue under this limit is suppressed [7,23,33]. Moreover, Ref. [3] many polymers show a similar nonlinear S-N curve (fatigue stress amplitude vs. number of fatigue cycles) to metals, indicating that the Basquin relation (high-cycle fatigue, HCF) and Coffin–Manson (low-cycle fatigue, LCF) formula are applicable [36,37].

The article continues by introducing the fatigue damage model for polymers and the steps for the calibration of the model (based on the Basquin-Coffin–Manson formulas). Based on the proposed endurance function, a novel rule for quantifying the impact of mean and alternating stresses on the fatigue life (the Haigh diagram) is also proposed, and it can consider the asymmetry between compression and tension (an alternative to the celebrated Gerber’s rule (1874)). The capability of the approach under different cyclic loads for the technologically important polycarbonate (PC) polymer is addressed.

## 2. Material and Methods

**Figure 2:**
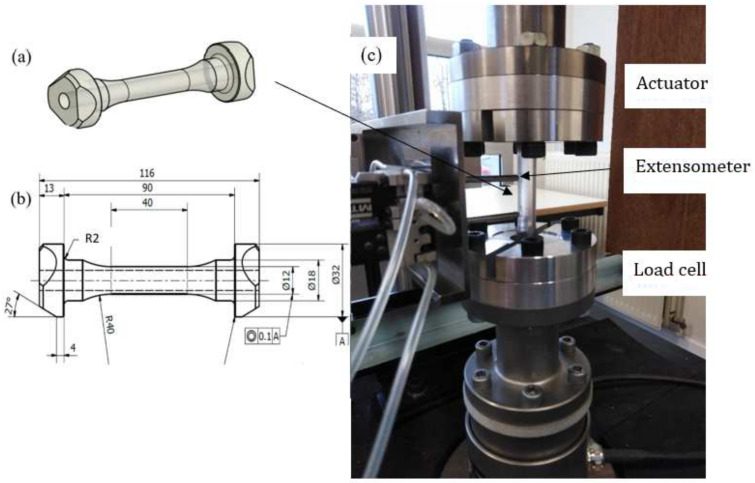
Geometryof the test specimen (**a**,**b**) and the testing equipment (**c**).

The PC polymer (Lexan^®^ 223R granulate, a density of 1.2 g/cm^3^) characterized by high impact and fatigue resistances was applied in the cyclic fatigue tests. The motivation of this material was that PC is one of the most commonly used polymers [1]. The geometry of the injection-molded flat (dog-bone shape) tensile specimen is in accordance with the standard (type IV specimen) [40]. The only deviation from the standard was that the gauge length was chosen to be 40 mm to obtain better compatibility with the extensometer, with its gauge length of 25 mm. A comparison was made with a tubular specimen’s geometry shown in Figure 2a,b, which is based on the standard [41]. The tubular geometry was used to avoid premature necking and buckling under tension and compression, respectively (the reduced inner diameter of 9 mm was applied to avoid premature necking and buckling). Optic 3D metrology (Alicona analyzer) was used to verify the excellent quality of the final shape of the specimens. The found surface faults were under 0.03 mm, and their influence on the test results was interpreted to be infinitesimal because of the inaccuracies they caused, for instance, in the outer diameter (12 mm) and cross-section of the gauge section of the tubular specimen were only 0.5% and 1.1%, respectively.

### Tests

The cyclic alternating (tension–compression) and pulsating (tensile) tests according to the standard [42] were conducted by applying an Instron^®^ Electropulse E10000 test machine with a load capacity of 10 kN and a displacement capacity of ±30 mm:

- cyclic fatigue tests at f=5 Hz (sinusoidal, force-control) until rupture at the stress ratios R=−1, R=0.1, and 0.5. The maximum stresses were 25, 35, and 50% of the rupture (ultimate) stress, 60 MPa, for R=−1, 15, 37.5, 50, 75, 90, and 97% for R=0.1, and 37.5, 50, 75, 90, and 97% for R=0.5.

The test set setup was designed to investigate the influence of the stress amplitude and mean stress on the deformation (ratcheting) and fatigue life. The axial elongation *u* and the corresponding force *F* were recorded by the testing machine. In addition, the elongation was measured by an extensometer (Instron 2620.601, capacity of 20% strain) glued onto the surface of the specimens’ web, cf. Figure 2c. Data acquisition was 1000 Hz. The strain was defined as $\epsilon :=u/L_{g}$, where $L_{g}$ is the gauge length of the extensometer. The nominal (1st Piola–Kirchhoff) stress σ=F/A, where *A* is the original cross-sectional area of the gauge section (e.g., $A=\pi (d_{o}^{2}-d_{i}^{2})/4$ for the tubular specimens), was used. The error in relation to the Cauchy or true stress was small because the strains were rather small (less than 10%). However, the observed deformation behavior was plastic because the stress-strain σ−ϵ relationship was nonlinear, and ratcheting was observed. Also, scanning electron microscope (SEM) imaging (FEI Quanta 450 W EDS EDAX) was performed from the surface of the gauge section of the specimens to observe the micromechanical mechanisms and progress of fatigue failure at different stages before final rupture (after interrupted tests at 500, 1500, and 3500 cycles for R=0.1 and 75% of the ultimate stress, 60 MPa).

## 3. Theory-Modeling

### 3.1. Kinematics and Constitutive Theory

To allow notable deformations to be investigated, the applied model is founded on the multiplicative decomposition of the deformation gradient [7], i.e.,

(1) $$F=F^{e}F^{\mathrm{vep}},$$

where $F^{e}$ and $F^{\mathrm{vep}}$ define the local deformation due to the elastic and viscoelastic–plastic mechanisms of the chain network, respectively (cf. the Kröner–Lee decomposition, $F^{e}F^{p}$, for the elastic-plastic mechanical behavior). Whereas the elastic part represents the reversible elastic mechanisms of the chain network, the viscoelastic–plastic part denotes the partially reversible mechanisms of chains, macroscopically corresponding to the recovery of strain after a stress removal (viscoelasticity) and long-term creep strain and stress relaxation (viscoplasticity) [7,43]. The plastic effect is due to irreversible, dissipative mechanisms, such as chain breakage and the slippage of chain entanglements [13,43,44,45] due to the growth and coalescence of nano/microscale voids [7,23,46,47].

**Figure 3:**
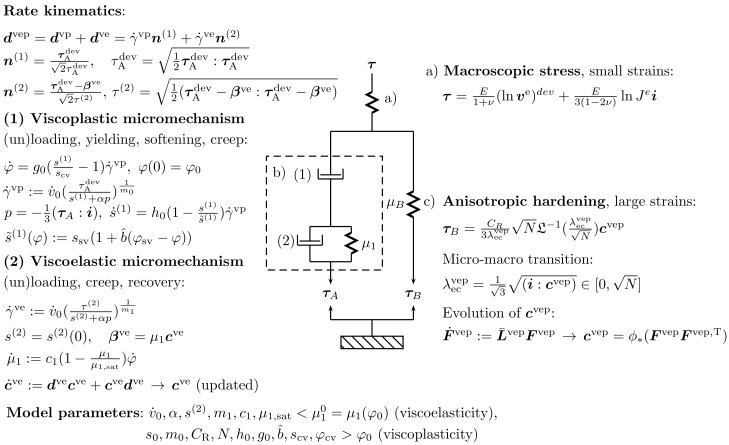
Representation of the constitutive model [7]. The model is governed by the following elements: (**a**) an elastic spring, (**b**) a nonlinear element (a dashpot (1) in series with a Kelvin–Voigt-like element (2)), and (**c**) a nonlinear Langevin spring. Evolution equations for the internal variables $s^{(}1)$ (shear resistance), φ (free volume), and $\mu_{1}$ (backstress modulus) are shown.

In the applied constitutive model introduced in [7,22], the elastic portion of the deformation is described by a single element (a) clearly separated from viscoelastic–plastic elements (b) and (c) in accordance with the classical [48] model, cf. Figure 3. As a result, the viscoplastic nonlinear Langevin spring (c) applied in the model to describe the anisotropic hardening of amorphous network structures (in large strains) is modeled solely using the viscous deformation driven by the backstress $\tau_{B}$ (as originally proposed in [49] for three dimensions). Moreover, the specified stress $\tau_{A}$ in Figure 3 defines the stress in each viscoelastic–plastic micromechanism.

**Figure 4:**
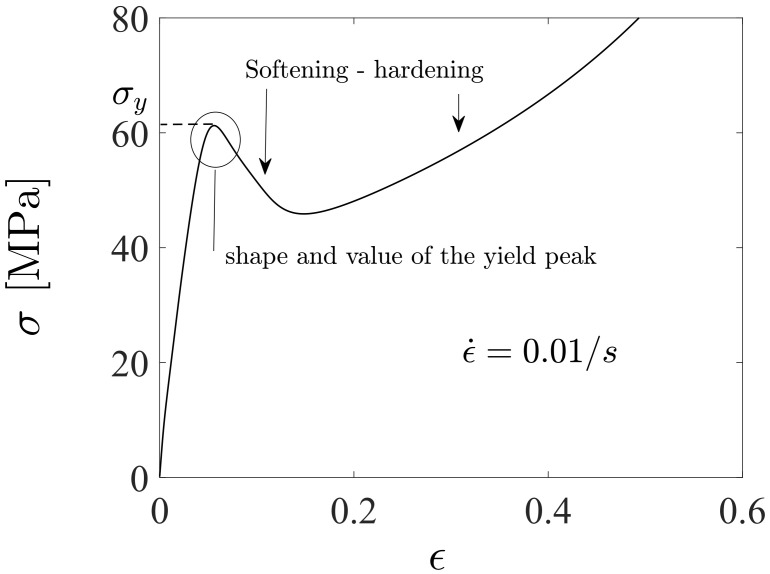
Experimentally observed, typical macroscopic stress vs. large strain curve for the applied polycarbonate. The peak yield stress $\sigma_{y}$ before softening is slightly more than 60 MPa.

The applied constitutive model is governed by the internal variables, $s^{\left(1\right)}$ (shear resistance), φ (average nano-to microscopic free volume), and $\mu_{1}$, cf. Figure 3. Considering the macroscopic stress vs. large strain relationship shown in Figure 4 (including softening followed by hardening [49]). First, the backstress modulus $\mu_{1}$ defines the slope of the initial response when the viscoelastic–plastic deformations start governing the macroscopic deformation behavior (σ varies between 20 and 30 MPa). The second internal model variable φ influences the pre-peak slope (before the yield peak) through the third internal model variable $s^{\left(1\right)}$, which also influences the peak yield stress and its post-peak slope. It was observed that the free volume φ (driven by the $\tau_{A}$) is one that strongly influences the shape and value of the yield peak, whereas the backstress $\tau_{B}$ influences the hardening in large strains before material rupture. The shape and value of the yield peak are of major importance when investigating low-cycle fatigue.

The proposed constitutive model is restricted to amorphous glassy polymers (thermoplastics without crystallization). A detailed account for the kinematics and constitutive theory of the model, including a numerical procedure, was discussed in [7] and, including the applied internal variables (φ, $s^{\left(1\right)}$, $\mu_{1}$), in [22]. For convenience, the rheological representation of the constitutive model, including the internal variables and material parameters applied, is illustrated in Figure 3.

### 3.2. Prediction of Fatigue Life

Based on the loading intensity, three ranges of fatigue life can be classified: ultra-low-, low-, and high-cycle ranges [30,50,51]. In the HCF region, the macroscopic deformation behavior of the material is primarily elastic, whereas in the ultra-low and LCF regions, notable plastic deformation evolves. The number of cycles during which the transition from LCF to HCF develops has been reported to range from a few thousand to tens of thousands, whereas ultra-LCF ranges from tens to hundreds of cycles. Engineering components, such as high-pressure pump housings, shafts, and gears for power transmission, when subjected to abnormally high loadings, are examples of components and structures subjected to ultra-LCF loadings [50,52,53].

**Figure 5:**
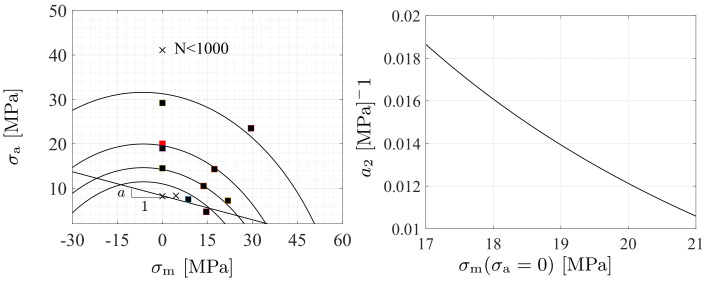
The Haigh diagrams for 40,000, 25,000, 13,000, and 5000 cycles (**left**). Markers *■* are the data points, and x are the points used in the model calibration. The red marker for data of a slightly different test specimen and PC is taken from [56]. The linear approximation $g∼aI_{1}$ ($I_{1}=\sigma_{m}$) for HCF (endurance limit) is also shown. The parameter $a_{2}$ vs. $\sigma_{m}$ (N=40,000) (**right**).

The proposed fatigue model governs all the fatigue regimes, and it is an annex of an appealing HCF model proposed by [28]. Although this approach is aimed for use in the context of metal fatigue, essentially characterized by dislocation micromechanisms, its formulation is rather general and suitable for various ductile solids that macroscopically show many similar fatigue characteristics [9,43,54]. The most important property is the macroscopical asymptotical extremes of lifetimes, i.e., the endurance limits can be determined, and fatigue under this limit is attenuated [9,23,34,55]. To generalize the endurance limit, the proposed fatigue model makes use of an endurance surface in the stress space. If the stress state is inside the endurance surface, no fatigue damage develops, whereas damage may develop if the stress state is outside the endurance surface. According to the proposed model, the endurance function representing the endurance surface is defined as

(2) $$\beta =\left(\bar{\sigma }+g\left(I_{1};a,a_{2}\right)-\sigma_{0}\right)/\sigma_{0}$$

wherein $\sigma_{0}$ is the fatigue strength limit or the endurance limit (experimentally observed stress amplitude for the alternating stress R=−1 under which fatigue damage does not develop) and the effective stress is

(3) $$\begin{array}{cc} \bar{\sigma } & =\sqrt{\frac{3}{2}\left(s-\alpha \right):M:\left(s-\alpha \right)}, \end{array}$$

where $s=\sigma -1/3I_{1}i$ is the deviatoric component of the stress tensor (the fatigue model is given in terms of the Cauchy stress σ=1/Jτ), $I_{1}=\mathrm{trace}\left(\sigma \right)=\sigma :i$ is the first stress invariant, and i is the identity tensor. The coefficient matrix M is defined as

(4) $$\left(s-\alpha \right):M:\left(s-\alpha \right)={\left(s-\alpha \right)}_{ii}^{2}+M{\left(s-\alpha \right)}_{ij}^{2},$$

i≠j=1,2,3; *M* is a material parameter. The coefficient matrix M allows the adjustment of the relationship between the uniaxial and torsional (shear) stresses, and its aim is to improve fatigue prediction under shear. In the original model by [28], low uniaxial stress states were investigated, and M was considered identity therein. Moreover, the linear relation $aI_{1}$ in the [28] model is replaced by the nonlinear function $g\left(I_{1};a,a_{2}\right)=\left(a+a_{2}I_{1}\right)I_{1}$ to encompass a large range of mean stresses as demonstrated in Figure 5(left). Using the steps in ([28], Section 4), it can be shown that the endurance surface (2) in a cyclic uniaxial loading (the stress varies between $\sigma_{m}-\sigma_{a}$ and $\sigma_{m}+\sigma_{a}$, where $\sigma_{m}$ and $\sigma_{a}$ are the mean stress and stress amplitude) reduces to the form

(5) $$\sigma_{a}^{2}+\frac{\sigma_{a}}{a_{2}}+\sigma_{m}^{2}+\frac{a}{a_{2}}\sigma_{m}-\frac{\sigma_{0}}{a_{2}}=0,$$

or equivalently

(6) $$\begin{array}{c} {\left(\sigma_{a}+\frac{1}{2a_{2}}\right)}^{2}+{\left(\sigma_{m}+\frac{a}{2a_{2}}\right)}^{2}=K=\frac{1+a^{2}+4a_{2}\sigma_{0}}{4a_{2}^{2}} \end{array}$$

which formula represents the Haigh diagram for polymers and is an alternative to the celebrated Gerber’s rule (1874) for ductile materials (inaccurate to predict the asymmetry between tension and compression). When $\sigma_{m}=0$ (R=−1), Equation (5) provides a small safety margin between $\sigma_{a}$ and $\sigma_{0}$ ($\sigma_{a}<\sigma_{0}$). This difference is motivated by the fact that the determination of the final value of $\sigma_{0}$ requires very time-consuming and costly tests, i.e., the Formulas (5) and (6) take this concern automatically into account. The solution of (5) is

(7) $$\sigma_{a}=\frac{1}{2a_{2}}\left(\sqrt{1+4a_{2}\left(\sigma_{0}-a\sigma_{m}-a_{2}\sigma_{m}^{2}\right)}-1\right).$$

A conclusion is that the first stress invariant $I_{1}$ in the endurance function (2) reflects the effect of the hydrostatic stress, $I_{1}/3$: the hydrostatic tension promotes the fatigue accumulation whereas fatigue is attenuated under hydrostatic compression, Figure 5(left). It should be mentioned that the Haigh diagrams do not reach the same point on the ultimate of the horizontal axis as the conventional metals do [28]. The positive parameter a∼0.2 defines in uniaxial fatigue loadings the initial (negative) slope of the Haigh diagram. Furthermore, selecting $\sigma_{m}\left(\sigma_{a}=0\right)={\bar{\sigma }}_{m}$, the solution (7) provides that $\sigma_{0}-a{\bar{\sigma }}_{m}-a_{2}{\bar{\sigma }}_{m}^{2}=0$ or

(8) $$a_{2}=\frac{\sigma_{0}-a{\bar{\sigma }}_{m}}{{\bar{\sigma }}_{m}^{2}}.$$

Because $a_{2}$ is constant, one is free to select the Haigh diagram of almost $\sigma_{0}$ for the HCF-limit, which is illustrated in Figure 5(right).

**Figure 6:**
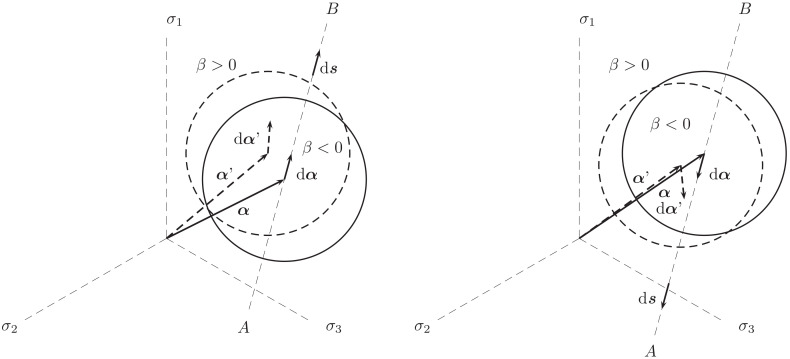
Movement of an endurance surface in deviatoric stress plane under a fluctuating loading (possibly non-proportional) (**left**) and unloading (**right**). The endurance surface reaches the current stress point and then starts to move between the states *A* and *B* (not necessarily fixed [28]). Peripheries of the surfaces in the current and an initial state are highlighted by the solid and dashed curves, respectively. Marking d signifies a small increment.

The endurance surface, as demonstrated in Figure 6, is spherical in the deviatoric plane, and it is the α tensor that determines the midpoint. The evolution of α is described by

(9) dα=C(s−α)dβ

where *C* is a material parameter [28]. Once a fatigue loading is actuated, the endurance surface can track the current stress due to the movement of α, which is, according to (9), always in the direction of s−α [28], see Figure 6. The evolution Equation (9) further reveals that the α tensor can memorize the load history because its evolution allows the value β=0, i.e., the movement of the endurance surface in the stress space. It is solely postulated that fatigue damage *D* and the backstress α, which is an overall force for *D*, only evolve on or outside the endurance surface (β≥0) and only when dβ>0, i.e., when the stress has crossed the surface and recedes from it, cf. [28]. This situation is demonstrated in Figure 6.

**Proposition** **1.** *The fatigue damage D only develops under the conditions*

(10) β≥0,dβ>0⇒dD>0,dα≠0.

#### Fatigue Damage Evolution

When dealing with fatigue from low to high cycles, an appropriate damage rule, in addition to the constitutive (plasticity) model, composes an integral part of the approach and analysis. The damage evolution is computed by exploiting an evolution law based on a scalar-valued damage variable. Description of fatigue damage by a scalar is valid because it solely represents the formation of microcracks in average (macroscopically) that, most of all, typically governs a majority of the total fatigue life (over 90% for polymers) [7,33,34,57,58,59].

Let then $x_{0}$ and $t_{0}$ be the initial material placement and initial time, and $x_{b}$ and $t_{b}$ the final critical placement and critical time instant for critical fatigue failure (over 90% of the total fatigue life), respectively. Then, damage at an initial instant is $D\left(x_{0},t_{0}\right)=D_{0}∼0$, and a local fatigue failure instantaneously prior to a notable progress of small cracks to a form of large cracks is given by $D\left(x_{b},t_{b}\right)=D_{b}∼1$ (the error in fatigue life is less than 10%). Since damage never decreases, based on Proposition 1 (dβ>0), an exponential expression,

(11) $$dD=\frac{K}{1-BD}\exp\left(F\left(\beta ;L,\tilde{L},\kappa \right)\right)d\beta \geq 0$$

where *B*, *K*, κ, *L*, and $\tilde{L}$ are positive parameters and are valid for the damage evolution. The parameter *B* defines the final asymptote of damage prior to a rapid macrostructural rupture (after 90% of the fatigue life [23,34]). Since the final damage evolution is rapid, the value of *B* needs to be sufficiently large (>0.5).

Many polymers show an endurance limit that is remarkably lower than the fatigue strength under LCF loads, and fatigue under this limit is attenuated [29,58,60]. Moreover, polymers exhibit only a small accumulation of fatigue strength as the cyclic lifetime decreases in the ultimate of the LCF region. To capture these charasteristics, a function F with two distinct linear asymptotes for a β-function is defined, i.e.,

(12) $$F\left(\beta ;L,\tilde{L},\kappa \right):=\left[L-\frac{{\tilde{L}}^{2}}{\kappa \beta }\left(\exp(-\frac{\kappa }{\tilde{L}}\beta )-1\right)\right]\beta .$$

**Figure 7:**
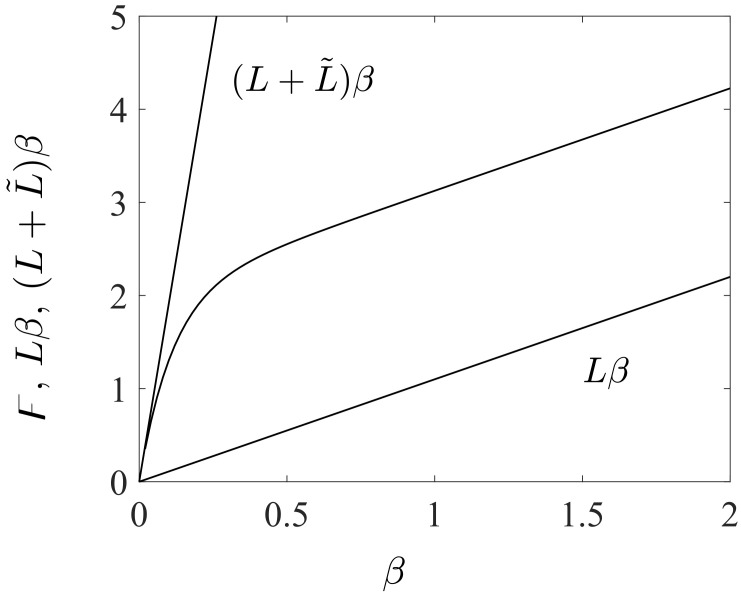
Function F and the asymptotes Lβ and $(L+\tilde{L})\beta$ for the LCF and HCF, respectively.

The function F has the asymptote $(L+\tilde{L})\beta$ as β is near zero in the ultimate of the HCF region and the expression Lβ when β>1 is large in the ultimate of the LCF region ($\kappa >>\tilde{L}$ or κ is the magnitude greater than $\tilde{L}$), see Figure 7. The curvature κ completes the definition of how rapidly the transition between the asymptotes is reached. Using F (12) in (11) results in the following multiplicative composition of the damage evolution:

(13) $$\begin{array}{c} dD=\frac{K}{1-BD}\exp\left(L\beta \right)\exp\left(-\frac{{\tilde{L}}^{2}}{\kappa }\left(\exp(-\frac{\kappa }{\tilde{L}}\beta )-1\right)\right)d\beta , \end{array}$$

where the first and latter exponential functions represent the fatigue damage evolution in the HCF and LCF regions, respectively. The numerical integration of the evolution Equations (9) and (13) is discussed in the Appendix A. The proposed continuum mechanics framework including damage is unified and consistent as it contains stress-based fatigue limits and accumulation of damage for arbitrary stress histories without a material-based constitutive theory, i.e., *D* is not e.g., a measure of the loss of stiffness ((1−D)E) [61,62], but it represents the ductile formation of microcracks on average (macroscopically) that governs a majority of the total fatigue life (over 90% for polymers) as has been demonstrated by the experiments (*D* and the Young´s modulus *E* can be regarded as uncoupled) [7,33,34,57,58,59].

### 3.3. Model Calibration

#### 3.3.1. Constitutive Model Parameters

The model was implemented using the Intel^®^ Fortran application. The constitutive variables shown in Figure 3 were regarded as uncoupled from fatigue, i.e., the evolution Equations (9) and (11) for fatigue were solved once the constitutive variables were known. This treatment is motivated by the experimental observations showing that the fatigue damage represents essentially the long-term formation of microcracks that typically covers the majority of the fatigue life (over 90%) and that has not a distinct influence on the macroscopic deformations and stresses [7,33,34,58] (the remaining fatigue life, ∼10% when the constitutive variables and parameters (Young’s modulus, *E*) and fatigue damage *D* must be regarded as coupled, is not significant for applications in practice).

The measurement of the elastic component $F^{e}$ in the decomposition of the deformation gradient (1) under force-controlled uniaxial (fatigue) loadings (for the model calibration) is straightforward: divide the measured uniaxial stress with the observed Young’s (elastic) modulus. Then, based on the generalized Hooke’s law (in three dimensions), the well-known Poisson’s ratio is applied to define other components of $F^{e}$. Instead, owing to path dependency of the viscoelastic–plastic deformation, $F^{\mathrm{vep}}$, its observation is very challenging, and therefore, it is just a kinematic model measure, solved numerically. A detailed discussion of the numerical treatment and calibration of the constitutive model in relation to the data was discussed in [7,22]. The constitutive model parameters used in the predictions are given in Table 1 and Table 2.

#### 3.3.2. Fatigue Model Parameters

**Figure 8:**
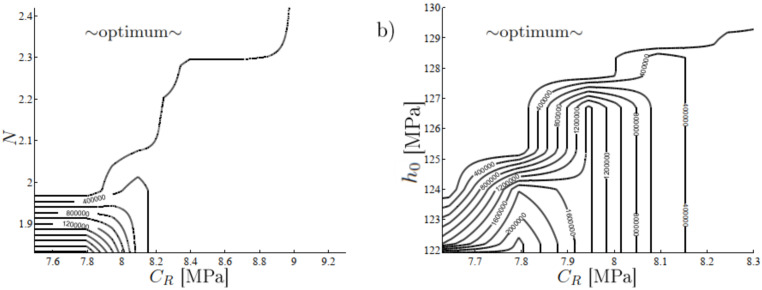
The weightedstress object, $\tilde{\sigma }=\Sigma_{i=1}^{\tilde{N}}\left(\psi ∥{\bar{\sigma }}_{UC,i}-\sigma_{UC,i}{∥}_{2}+{∥{\bar{\sigma }}_{PSC,i}-\sigma_{PSC,i}∥}_{2}\right)$ as a function of the constitutive parameters (**a**) $C_{R}$ and *N* and (**b**) $C_{R}$ and $h_{0}$ (see Figure 3). The bar indicates the experimental stress response (extracted from [65]), $\tilde{N}$ is the number of stress increments (up to rupture), and ψ=5 is the weight factor. The fitting is performed through uniaxial (UC) and plane strain compression (PSC) modes taken the two strain rates $\dot{\epsilon }=0.001\;s^{-1}$ and $\dot{\epsilon }=0.01\;s^{-1}$ into account.

The model parameters for fatigue were extracted from uniaxial in situ measurements (smooth specimen) for the applied stress vs. number of cycles (S−N curves). The parameters a∼0.2, $a_{2}$∼0.015, $\sigma_{0}$∼9 MPa, and *B*∼0.7, were defined above, whereas the remaining parameters are more complicated because they are related to the intrinsic development of the high-cycle damage (*K*∼$10^{-5}$, *L*∼1…3), backstress α (*C*∼1) [28], and deformation history of the low-cycle damage ($\tilde{L}$, κ). These parameters can be determined using a heuristic (trial-and-error) iteration, clustering of scattered parameters [63], least-squares fitting, or an optimization procedure [7,21,64]. Then, however, parameters do not have a clear physical or mechanical interpretation, and they may show different optimal values depending on the applied object function. An example is demonstrated in Figure 8, which shows that the stress response is highly nonlinear on the part of constitutive parameter spaces and almost zero elsewhere. Consequently, the tolerance for the optimum is achieved on different parameter values, i.e., the optimum is not unique or difficult to find.

The S-N curves of polymers exhibit a fairly straight slanting portion with a negative slope at low numbers of cycles and a virtually horizontal line in high cycles when the maximum stress equals to the endurance limit. Therefore, many polymers show a nonlinear S-N curve similar to metals [29], indicating the well-defined Basquin relation (HCF) and Coffin–Manson (LCF) formula are applicable [36,37].

**Figure 9:**
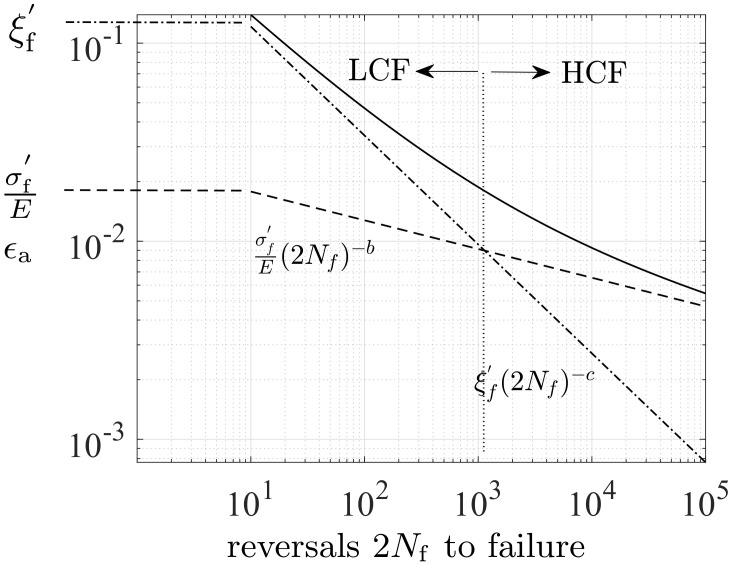
Strain amplitude vs. fatigue life based on the Basquin (HCF) and Coffin–Manson (LCF) formulas.

The interpretation of the Basquin and Coffin–Manson formulas is evident from the strain amplitude under uniaxial fully reversed fatigue loads (R=−1):

(14) $$\frac{\Delta \epsilon }{2}=\frac{\Delta \epsilon^{p}}{2}+\frac{\sigma_{a}}{E}=\xi_{f}^{{}^{\prime }}{\left(2N_{f}\right)}^{-c}+\frac{\sigma_{f}^{{}^{\prime }}}{E}{\left(2N_{f}\right)}^{-b},$$

where $\xi_{f}^{{}^{\prime }}$ (fatigue ductility coefficient), *c* (fatigue ductility exponent), $\sigma_{f}^{{}^{\prime }}$ (fatigue strength coefficient), and *b* (fatigue strength exponent) are parameters ([51,66] page 678, Equation (9)), see Figure 9. Despite the similar fatigue characteristics between polymers and metals, there is no data for these parameters for polymers. The steps to define these parameters for polymers are also discussed next.

#### Polymer vs. Metal Fatigue Parameters

Considering a periodic uniaxial loading with sufficiently low mean stress ($\sigma_{m}\in \left(-5,15\right)$ MPa, see Figure 5(left)), the function *g* in (2) and the effective stress (3) are reduced to $g∼aI_{1}$ and

(15) $$\bar{\sigma }=k\left(\sigma -3/2\alpha \right),\;\;k=1\;\;if\;\;\sigma -3/2\alpha >0\;\;and\;\;k=-1\;\;otherwise,$$

respectively. The endurance function (2) can then be given as

(16) $$\beta =\frac{1}{\sigma_{0}}\left(k\left(\sigma -3/2\alpha \right)+a\sigma -\sigma_{0}\right),$$

cf. [28]. Turning to the evolution Equation (9) for the backstress α and taking advantage of (16),

(17) $$d\alpha =\frac{2C}{3\sigma_{0}}\left(k\left(d\sigma -3/2d\alpha \right)+ad\sigma \right)\left(\sigma -3/2\alpha \right).$$

**Figure 10:**
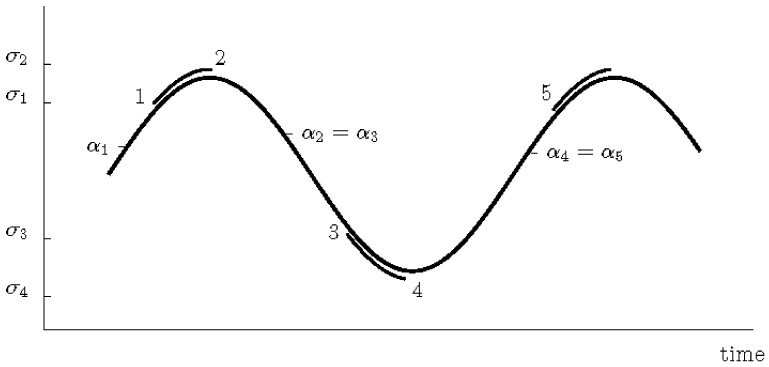
Periodic uniaxial sinusoidal stress state. Simultaneous damage development and movement of the endurance surface are designated by a double curve.

The stress is presumed to alter periodically between $\sigma_{2}=\sigma_{m}+\sigma_{a}$ and $\sigma_{4}=\sigma_{m}-\sigma_{a}$, see Figure 10, and the stresses $\sigma_{1}$ and $\sigma_{3}$ are the stress states on the endurance surface, where $\beta_{1}=\beta_{3}=0$.

#### HCF Region

Considering first the HCF model parameters (*L*, *C*, $\bar{K}$). Noting β is near zero in the HCF region, integration of the damage evolution (13) from state 1 to state 2 yields

$$D_{2}-D_{1}=\frac{\bar{K}}{L}\left(\exp\left(L\beta_{2}\right)-\exp\left(L\beta_{1}\right)\right),$$

where $\bar{K}=K/\left(1-BD\right)∼K/0.7$ holds for most of the fatigue life (B∼0.7 and 0.2<D<0.5 for most of the fatigue life). Observing that state 1 is located on the endurance surface, i.e., $\beta_{1}=0$,

$$D_{2}-D_{1}=\frac{\bar{K}}{L}\left(\exp\left(L\beta_{2}\right)-1\right),$$

cf. [28]. Likewise, integration from state 3 to 4 yields

$$D_{4}-D_{3}=\frac{\bar{K}}{L}\left(\exp\left(L\beta_{4}\right)-1\right).$$

The damage evolution during one complete cycle becomes

(18) $$\Delta D=\left(D_{2}-D_{1}\right)+\left(D_{4}-D_{3}\right)=\frac{\bar{K}}{L}\left(\exp\left(L\beta_{2}\right)+\exp\left(L\beta_{4}\right)-2\right).$$

Using (16) in (18), it follows that during *N* cycles to failure (D=1)

(19) $$\frac{L}{\bar{K}N}=\exp\left(\frac{L}{\sigma_{0}}\left(k\left(\sigma_{2}-\frac{3}{2}\alpha_{2}\right)+a\sigma_{2}-\sigma_{0}\right)\right)+\exp\left(\frac{L}{\sigma_{0}}\left(k\left(\sigma_{4}-\frac{3}{2}\alpha_{4}\right)+a\sigma_{4}-\sigma_{0}\right)\right)-2.$$

Two standard testing loads are examined: alternating, R=−1, and pulsating, R=0 [29]. When R=−1, $d\sigma_{4}=d\sigma_{2}=0$, $\sigma_{4}=-\sigma_{2}$, and Equation (17), at states 2 and 4, provides

(20) $$d\alpha_{2}=\frac{2C}{3\sigma_{0}}\left(-3/2kd\alpha_{2}\right)\left(\sigma_{2}-3/2\alpha_{2}\right),$$

(21) $$d\alpha_{4}=\frac{2C}{3\sigma_{0}}\left(-3/2kd\alpha_{4}\right)\left(-\sigma_{2}-3/2\alpha_{4}\right).$$

The solutions of (20) and (21) are

(22) $$\sigma_{2}-3/2\alpha_{2}=\frac{\sigma_{0}}{C},\;\;k=-1,$$

and

(23) $$-\sigma_{2}-3/2\alpha_{4}=\frac{\sigma_{0}}{C},\;\;k=-1.$$

Substitution of (22) and (23) into (19) results in

(24) $$\frac{L}{\bar{K}N}=\exp\left(\frac{L}{\sigma_{0}}\left(a\sigma_{2}+\frac{\sigma_{0}}{C}-\sigma_{0}\right)\right)+\exp\left(\frac{L}{\sigma_{0}}\left(-a\sigma_{2}+\frac{\sigma_{0}}{C}-\sigma_{0}\right)\right)-2.$$

It is compulsory that $L/(\bar{K}N)\to 1$ as $\sigma_{2}\to \sigma_{0}$ and R=−1. Then, from (24), one obtains after rearrangements (noting exp(La)+exp(−La)∼2):

(25) $$C=\frac{L}{L+\ln(3/2)}.$$

When R=0, $d\sigma_{4}=d\sigma_{2}=0$, $\sigma_{4}=0$, and using (17), in a similar way as above, it follows that

(26) $$\sigma_{2}-3/2\alpha_{2}=\frac{\sigma_{0}}{C},\;\;k=-1,$$

and

(27) $$-3/2\alpha_{4}=\frac{\sigma_{0}}{C},\;\;k=-1.$$

Substitution of (26) and (27) into (19) results in

(28) $$\frac{L}{\bar{K}\bar{N}}=\exp\left(\frac{L}{\sigma_{0}}\left(a\sigma_{2}+\frac{\sigma_{0}}{C}-\sigma_{0}\right)\right)+\exp\left(\frac{L}{\sigma_{0}}\left(\frac{\sigma_{0}}{C}-\sigma_{0}\right)\right)-2,$$

where $\bar{N}$ is the number of cycles to fatigue failure when R=0. Using $\sigma_{2}=2\sigma_{a}=2\sigma_{m}=2\zeta \sigma_{0}$ in (28) yields (noting exp(2Laζ)+1∼exp(2Laζ+1/2))

(29) $$C=\frac{L}{L\left(1-2a\zeta \right)+\ln\left(\frac{L}{\bar{K}\bar{N}}+2\right)+1/2}.$$

A comparison of (25) and (29) yields

(30) $$L=\frac{\ln(\left(\frac{L}{\bar{K}\bar{N}}+2\right)/3)+1/2}{2a\zeta }.$$

The Basquin equation for HCF is $\sigma_{0}=\sigma_{f}^{{}^{\prime }}{\left(2N\right)}^{-}b$, where b and $\sigma_{f}^{{}^{\prime }}$ are positive material parameters [29]. For R=0, when $\bar{N}$ is the number of cycles for failure (D=1), the relation $\sigma_{f}^{{}^{\prime \prime }}{\left(2\bar{N}\right)}^{-}b=\sigma_{f}^{{}^{\prime }}{\left(2N\right)}^{-}b$ yields

(31) $$\frac{1}{\bar{N}}={\left(\frac{\sigma_{f}^{{}^{\prime }}}{\sigma_{f}^{{}^{\prime \prime }}}\right)}^{1/b}\frac{1}{N}.$$

Certain enhancements have been developed to the Basquin formula for the effect of mean stress, R≠−1 ($\sigma_{m}\neq 0$, ζ≥1) [67]:

(32) $$\sigma_{f}^{{}^{\prime \prime }}=\varpi^{-1}\sigma_{f}^{\prime }\exp\left(-{\left(\frac{\sigma_{m}}{\sigma_{u}}\right)}^{\sigma_{\mathrm{yi}}/\sigma_{u}}\right),$$

where $\sigma_{m}$, $\sigma_{\mathrm{yi}}$, and $\sigma_{u}$ are the mean, yield, and ultimate stresses, respectively [67], and ϖ<1∼0.9 accounts for a slightly reduced elastic portion of strain $\sigma_{f}^{{}^{\prime \prime }}/\left(E/\varpi \right)$ for polymers when the mean stress relative to the stress amplitude increases ([7], Figures 13 and 14), cf. Figure 9. Using (32) in (30) and (31) yields (noting $L/(\bar{K}N)\to 1$)

(33) $$L=\frac{\ln(\left(\varpi {\left(\exp\left({\left(\frac{\sigma_{m}}{\sigma_{u}}\right)}^{\sigma_{\mathrm{yi}}/\sigma_{u}}\right)\right)}^{1}/b+2\right)/3)+1/2}{2a\zeta }.$$

Finally, making use of the relation $\sigma_{0}=\sigma_{f}^{{}^{\prime }}{\left(2N\right)}^{-b}=\sigma_{f}^{{}^{\prime }}{\left(2L/\bar{K}\right)}^{-b}$ yields

(34) $$\bar{K}=2L{\left(\frac{\sigma_{0}}{\sigma_{f}^{{}^{\prime }}}\right)}^{1/b}.$$

Equations (25), (33) and (34) result in the eligible expressions for the fatigue model parameters *C*, *L*, and *K*, that is, the proposed fatigue model relies on the well-defined parameters $\sigma_{u}$ and $\sigma_{y}$, and the values of the parameters $\sigma_{f}^{\prime }$ and b (Basquin), which are well defined and documented for metal fatigue [29].

#### LCF Region

The model parameters for the LCF are $\tilde{L}$ and κ. In the LCF region, β can vary largely and the integration of the damage evolution (13) from state 1 to state 2 yields

(35) $$\begin{array}{cc} D_{2}-D_{1} & =\bar{K}\exp\left(-\bar{c}\right)(\frac{1}{L}\left(\exp\left(L\beta_{2}\right)\exp\left(\bar{c}\exp\left(-\bar{b}\beta_{2}\right)\right)-\exp\left(\bar{c}\right)\right)+\frac{\bar{c}\bar{b}}{L(L-\bar{b})}(\exp\left(\left(L-\bar{b}\right)\beta_{2}\right)\cdot \\ & \;\exp\left(\bar{c}\exp\left(-\bar{b}\beta_{2}\right)\right)-\exp\left(\bar{c}\right))+\frac{{\left(\bar{c}\bar{b}\right)}^{2}}{L(L-\bar{b})}\int_{\beta_{1}}^{\beta_{2}}\exp\left(\left(L-2\bar{b}\right)\beta \right)\exp\left(\bar{c}\exp\left(-\bar{b}\beta \right)\right)), \end{array}$$

where the constraint $\beta_{1}=0$ was considered, and $\bar{b}=\kappa /\tilde{L}$ and $\bar{c}=-{\tilde{L}}^{2}/\kappa$. Since $\exp\left(L\beta_{2}\right)>>\exp\left(\left(L-\bar{b}\right)\beta_{2}\right)>>\exp\left(\left(L-2\bar{b}\right)\beta_{2}\right)$ ($\kappa >>\tilde{L}>L$, $\beta_{2}>0$) while the coefficients 1/L, $\bar{c}\bar{b}/L\left(L-\bar{b}\right)$, and ${\left(\bar{c}\bar{b}\right)}^{2}/L\left(L-\bar{b}\right)$ differs relatively slightly, Equation (35) is reduced to

(36) $$\begin{array}{cc} D_{2}-D_{1} & =\frac{\bar{K}}{L}(\exp\left(L\beta_{2}\right)\exp\left(\bar{c}\left(\exp\left(-\bar{b}\beta_{2}\right)-1\right)\right)-1). \end{array}$$

Likewise, integration from state 3 to 4 yields

$$D_{4}-D_{3}=\frac{\bar{K}}{L}(\exp\left(L\beta_{4}\right)\exp\left(\bar{c}\left(\exp\left(-\bar{b}\beta_{4}\right)-1\right)\right)-1).$$

The damage evolution during one cycle ($D=\left(D_{2}-D_{1}\right)+\left(D_{4}-D_{3}\right)$) becomes

(37) $$\Delta D=\frac{\bar{K}}{L}(\exp\left(L\beta_{2}\right)\exp\left(\bar{c}\left(\exp\left(-\bar{b}\beta_{2}\right)-1\right)\right)+\exp\left(L\beta_{4}\right)\exp\left(\bar{c}\left(\exp\left(-\bar{b}\beta_{4}\right)-1\right)\right)-2).$$

In the LCF region, the pulsating stress (R=0) is high, resulting in ratcheting [7]. This characteristic limits the capability of the Coffin–Manson formula for LCF. Therefore, considering solely the alternating stress (R=−1, $d\sigma_{4}=d\sigma_{2}=0$, $\sigma_{4}=-\sigma_{2}$). Using (16), (22), and (23) in (37), it follows that during $\tilde{N}$ cycles to fatigue failure (D=1):

(38) $$\begin{array}{cc} \frac{L}{\bar{K}\tilde{N}} & =\exp\left(-\bar{c}\right)(\exp\left(\frac{L}{\sigma_{0}}\left(a\sigma_{2}+\frac{\sigma_{0}}{C}-\sigma_{0}\right)\right)\exp\left(\bar{c}\left(\exp\left(\frac{-\bar{b}}{\sigma_{0}}\left(a\sigma_{2}+\frac{\sigma_{0}}{C}-\sigma_{0}\right)\right)\right)\right)+\\ & \exp\left(\frac{L}{\sigma_{0}}\left(a\sigma_{4}+\frac{\sigma_{0}}{C}-\sigma_{0}\right)\right)\exp\left(\bar{c}\left(\exp\left(\frac{-\bar{b}}{\sigma_{0}}\left(a\sigma_{4}+\frac{\sigma_{0}}{C}-\sigma_{0}\right)\right)\right)\right))-2. \end{array}$$

Using $\sigma_{2}=\bar{\zeta }\sigma_{0}$ ($\bar{\zeta }>1$ in LCF region), (38) becomes

(39) $$\begin{array}{cc} \frac{L}{\bar{K}\tilde{N}} & =\exp\left(-\bar{c}\right)(\exp\left(L\left(a\bar{\zeta }+\frac{1}{C}-1\right)\right)\exp\left(\bar{c}\left(\exp\left(-\bar{b}\left(a\bar{\zeta }+\frac{1}{C}-1\right)\right)\right)\right)+\\ & \exp\left(L\left(-a\bar{\zeta }+\frac{1}{C}-1\right)\right)\exp(\bar{c}\left(\exp\left(-\bar{b}\left(-a\bar{\zeta }+\frac{1}{C}-1\right)\right)\right))-2\\ & \approx \exp\left(-\bar{c}\right)\left(\exp\left(L\left(a\bar{\zeta }+\frac{1}{C}-1\right)\right)\exp\left(\bar{c}\left(\exp\left(-\bar{b}\left(a\bar{\zeta }+\frac{1}{C}-1\right)\right)\right)\right)\right)-2, \end{array}$$

because C∼1, $\bar{b}=\kappa /\tilde{L}>>1$, and $\bar{c}=-{\tilde{L}}^{2}/\kappa <0$. From (39), one obtains after rearrangements:

(40) $$\bar{b}=\frac{\kappa }{\tilde{L}}=\frac{-const_{2}}{a\bar{\zeta }+\frac{1}{C}-1},\;const_{2}=\ln(\frac{1}{\bar{c}}\left(\ln\left(\frac{L}{\bar{K}\tilde{N}}+2\right)-L\left(a\bar{\zeta }+\frac{1}{C}-1\right)+\bar{c}\right)).$$

The parameters κ and $\tilde{L}$ for LCF are yet undetermined. Therefore, considering third alternating stress $\sigma_{2}=\bar{\bar{\zeta }}\sigma_{0}$, $\bar{\bar{\zeta }}>\bar{\zeta }>1$ such that N<1000 for ultimately LCF. Then $\exp(-\bar{b}\beta_{2})∼0$ ($\bar{b}>>1$ and $\beta_{2}>1$), and the damage evolution (37) during one complete cycle further reduces to

(41) $$\Delta D=\frac{\bar{K}}{L}(\exp\left(-\bar{c}\right)\exp\left(L\beta_{2}\right)-2).$$

Using (16) in (41) and noting (22) and (23), it follows, during $\tilde{\tilde{N}}$ cycles to failure (D=1), that

(42) $$\frac{L}{\bar{K}\tilde{\tilde{N}}}=\exp\left(-\bar{c}\right)\exp\left(L\left(a\bar{\zeta }+\frac{1}{C}-1\right)\right)-2.$$

From (42), one obtains after rearrangements:

(43) $$C=\frac{L}{\ln\left(\frac{L}{\bar{K}\tilde{\tilde{N}}}+2\right)+\bar{c}+L\left(1-a\bar{\bar{\zeta }}\right)}.$$

A comparison of (25) and (43) yields

(44) $$\bar{c}=La\bar{\bar{\zeta }}-\ln\left(\frac{2}{3}\left(\frac{L}{\bar{K}\tilde{\tilde{N}}}+2\right)\right)=-const_{3}=-\frac{{\tilde{L}}^{2}}{\kappa }$$

or

(45) $$\tilde{L}=\sqrt{\kappa }\sqrt{const_{3}}.$$

Furthermore, a comparison of (40) and (45) reveals that

(46) $$\kappa ={\left(\frac{-const_{2}}{a\bar{\zeta }+\frac{1}{C}-1}\right)}^{2}const_{3}.$$

In the end, equalizing the Ramberg–Osgood (for dynamic stress-strain relations) and Coffin–Manson formulas for the plastic strain amplitude, i.e.,

$$\epsilon_{a}^{p}={\left(\frac{\sigma_{a}}{\sigma_{c}^{{}^{\prime }}}\right)}^{1/n_{c}}=\xi_{f}^{{}^{\prime }}{\left(2\tilde{N}\right)}^{-c},$$

results in

(47) $$\tilde{N}=\frac{1}{2}{\left[\frac{1}{\xi_{f}^{{}^{\prime }}}{\left(\frac{\sigma_{a}}{\sigma_{c}^{{}^{\prime }}}\right)}^{1/n_{c}}\right]}^{-1/c},\;\;\sigma_{a}=\bar{\zeta }\sigma_{0},$$

where $\sigma_{c}^{\prime }$ denotes the strength coefficient and $n_{c}$ is the strain hardening coefficient [29,66]. One can equalize (39) and (42) with negligible error in the LCF region by choosing $\bar{\bar{\zeta }}\to \bar{\zeta }$ ($\tilde{\tilde{N}}\to \tilde{N}$).

In summary, the fatigue model parameters are defined as follows:

(48) $$\begin{array}{c} \begin{array}{cc} L & =\frac{\ln(\left(\varpi {\left(\exp\left({\left(\frac{\sigma_{m}}{\sigma_{u}}\right)}^{\sigma_{\mathrm{yi}}/\sigma_{u}}\right)\right)}^{1/b}+2\right)/3)+1/2}{2a\zeta },\;\;\zeta \geq 1,\;\;\varpi ∼0.9,\\ C & =\frac{L}{L+\ln(3/2)},\\ \bar{K} & =2L{\left(\frac{\sigma_{0}}{\sigma_{f}^{{}^{\prime }}}\right)}^{1}/b,\;K=0.7\ldots 0.8\bar{K},\\ \kappa & ={\left(\frac{-const_{2}}{\frac{1}{C}+a\bar{\zeta }-1}\right)}^{2}const_{3},\\ const_{2} & =\ln(\frac{1}{-const_{3}}\left(\ln\left(\frac{L}{\bar{K}\tilde{N}}+2\right)-L\left(a\bar{\zeta }+\frac{1}{C}-1\right)-const_{3}\right)),\\ const_{3} & =-La\bar{\bar{\zeta }}+\ln\left(\frac{2}{3}\left(\frac{L}{\bar{K}\tilde{N}}+2\right)\right),\;\bar{\bar{\zeta }}\to \bar{\zeta }>\zeta \geq 1,\\ \tilde{L} & =\sqrt{\kappa }\sqrt{const_{3}}, \end{array} \end{array}$$

where $\tilde{N}$ is given by the Equation (47). It can be observed that the model parameters for fatigue of polymers are definable by the well-defined parameters $\sigma_{f}^{{}^{\prime }}$ (fatigue strength coefficient), *b* (fatigue strength exponent), $\xi_{f}^{{}^{\prime }}$ (fatigue ductility coefficient), *c* (fatigue ductility exponent), $\sigma_{c}^{{}^{\prime }}$ (strength coefficient), and $n_{c}$ (strain hardening coefficient) included in the Basquin, Coffin–Manson, and Ramberg–Osgood formulas [29,66].

## 4. Results

### 4.1. Fatigue Parameter Values and Sensitivity

The fatigue strength (endurance) limit for PC ranges between $\sigma_{0}=8\ldots 10$ MPa (R=−1) [68]. The parameter *a* is positive and represents the negative value of the slope in the Haigh diagram, see Figure 5(left), i.e., it can be calculated from the relation $a=(\sigma_{0}/\sigma_{+}0)-1$, in which $\sigma_{+}0$ is the fatigue strength for uniaxial tensile pulsating loading (R=0, $\sigma_{m}>0$). Using data for R=0.1∼0, $\sigma_{+}0=6.5\ldots 8.0$ MPa and then a=0.15…0.25. An extension $aI_{1}\to g$ in the endurance surface (2) to encompass large mean stresses is governed by the single parameter $a_{2}∼0.015$ (8) as illustrated in Figure 5(right).

**Figure 11:**
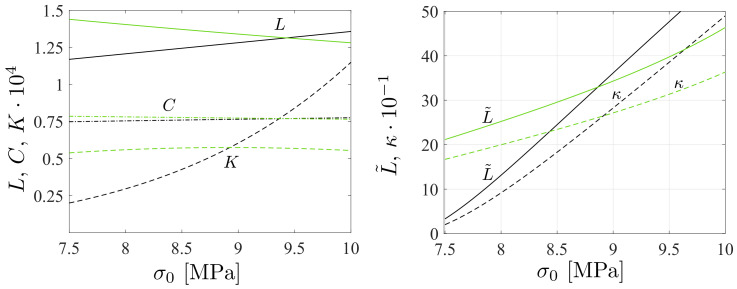
Fatigue parameters *C* (dash-dot), *L* (solid), *K* (dashed) (**left**), and $\tilde{L}$ (solid) and κ (dashed) (**right**). The black and green colors refer to the final parameter values and the parameter values depending on the range $\sigma_{0}=$7.5…10 MPa: $\sigma_{\mathrm{yi}}=34\ldots 42$ MPa, b=0.19…0.17, $\sigma_{f}^{{}^{\prime }}=55\ldots 58$ MPa (*C*, *L*; HCF), $\xi_{f}^{{}^{\prime }}=0.6\ldots 0.9$, c=0.54…0.62, $\sigma_{c}^{{}^{\prime }}=52\ldots 55$ MPa, and $n_{c}=0.08\ldots 0.04$ ($\tilde{L}$, κ; LCF).

To govern the ultimate fatigue life of the HCF region, ζ=1, i.e., $\sigma_{a}=\sigma_{0}$ for the stress ratios R=−1 ($\sigma_{m}=0$) and R=−0.3 ($\sigma_{m}=0.5\sigma_{a}$) was used to calculate *L*, *C*, and *K* defined in (48). These two points are shown in Figure 5(left). To find their exact values to also govern the intermediate region from LCF to HCF, the sensitivity of the Basquin model parameters (*b*, $\sigma_{f}^{{}^{\prime }}$) for HCF and the Coffin–Manson model parameters ($\xi_{f}^{{}^{\prime }}$, *c*) for LCF on them were investigated, see Figure 11(left). Due to the similar characteristics of the S-N curves between metals and polymers, the initial values of Basquin-Coffin–Manson model parameters for polymers were found from the values for metals [29]. The fatigue ductility coefficient $\xi_{f}^{{}^{\prime }}$ representing the ultimate of the strain amplitude $\epsilon_{a}$ (logarithmic) for the fatigue life of a few tens of cycles (R=−1) was approximated to be $\xi_{f}^{{}^{\prime }}<0.8$, as demonstrated in Figure 9. In this treatment, *c* (fatigue ductility exponent) defining the slope of the $\epsilon_{a}-N$ (S-N) curve in the LCF region was observed to vary 15% when $\sigma_{0}=7.5\ldots 10$ MPa varies 30%, see Figure 11. Moreover, the fatigue strength coefficient $\sigma_{f}^{{}^{\prime }}$ was considered to vary 6%, and the values close to the true tensile rupture (ultimate) stress, as have been used in the Basquin relationship for metals, were applied [29,66]. Based on the observations for R=−1, R=0.1, and R=0.5, the fatigue strength exponent *b* defining the slope of the $\epsilon_{a}-N$ (S-N) curve in the HCF region was observed to be virtually constant, and it is the parameter *K* most affected by *b*. In view of the results in Figure 11, the impact of the fatigue strength limit and the Basquin model parameters on the parameters *L* (weights the endurance function β in the rate of damage evolution, see (13) and Figure 7) and particularly *C* (influences the midpoint of the endurance surface, see (9) and Figure 6) is low.

**Figure 12:**
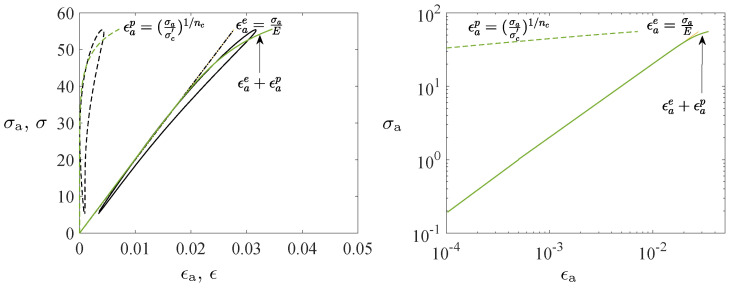
Stress vs. strain amplitudes based on the Ramberg–Osgood formula (green curves) for PC: linear scale (**left**) and logarithmic scale (**right**). The black curves (**left**) denote the experimental stress vs. strain responses, showing the nonlinear plastic behavior (dashed) and total deformation (solid).

To calculate κ and $\tilde{L}$ in (48) for LCF, $\bar{\zeta }\to \bar{\bar{\zeta }}=5.1$ (R=−1), i.e., $\sigma_{a}=\bar{\bar{\zeta }}\sigma_{0}∼1.1\sigma_{\mathrm{yi}}$ was used to result in N≤1000, as it was restricted above. This point is shown in Figure 5(left). In this treatment, $\sigma_{c}^{{}^{\prime }}$ (strength coefficient) and $n_{c}$ (strain hardening coefficient) applied in the Ramberg–Osgood formulas similar to metals were defined. The strength coefficient $\sigma_{c}^{{}^{\prime }}$ represents the strength when the material shows a significant yielding [66] and it was observed to be close to the fatigue strength coefficient $\sigma_{f}^{{}^{\prime }}$ used in the Basquin equation. The strain hardening coefficient $n_{c}$ defines the nonlinear relationship between the (plastic) strain and stress amplitudes, and in double logarithmic scale, when the relationship is linear, it defines the slope of the line as demonstrated in Figure 12(right). The variation of $n_{c}$ was 50 % (for $\sigma_{0}=7.5\ldots 10$ MPa), cf. Figure 11. In view of the results in Figure 11, the impact of the fatigue strength limit and the Ramberg–Osgood model parameters on the parameters $\tilde{L}$ (weights the endurance function β and influences the damage rate, see (13) and Figure 7) and κ (completes the damage evolution of how rapidly the transition between the LCF and HCF is reached, see (12)) is significant; the greater $\sigma_{0}$ and $\sigma_{c}^{{}^{\prime }}$, and the lower $n_{c}$, the greater $\tilde{L}$ (damage rate) and κ. This observation is consistent with our general experimental observations for LCF (stress levels near $\sigma_{y}$), which show more brittle and rapid damage evolution when the strength ($\sigma_{y}$, $\sigma_{0}$, $\sigma_{c}^{{}^{\prime }}$) and consequently the brittleness of the polymer material increase (many materials with brittle characteristics exhibit greater strength and hardness [69]).

**Figure 13:**
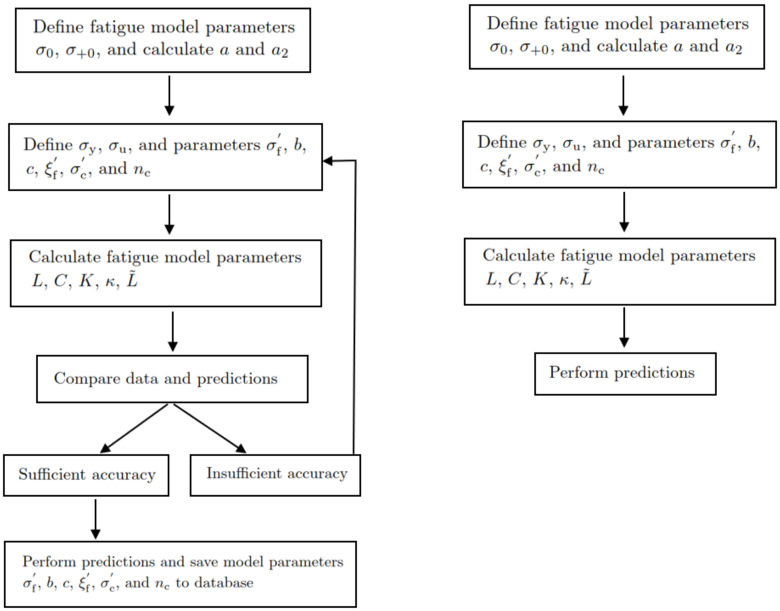
Flowcharts for model calibrations and predictions based on unavailable (**left**) and available (**right**) Basquin–Coffin–Manson and Ramberg–Osgood model parameters.

A conclusion is that the relationship between the fatigue model parameters (*L*, *C*, *K*, κ, $\tilde{L}$) and the Basquin, Coffin–Manson, and Ramberg–Osgood model parameters is highly nonlinear for polymers, but the relationship can be defined. The final parameter values to construct the S-N curves are shown in Table 3. An interesting detail is that the final parameter values for the Basquin-Coffin–Manson and the Ramberg–Osgood model parameters (in the title of Table 3) are almost the average values of their ranges; see the title of Figure 11. The proposed concept to define fatigue parameters *L*, *C*, *K*, κ, and $\tilde{L}$ for polymer fatigue is demonstrated in Figure 13.

### 4.2. Macrostructural Aspects

**Figure 14:**
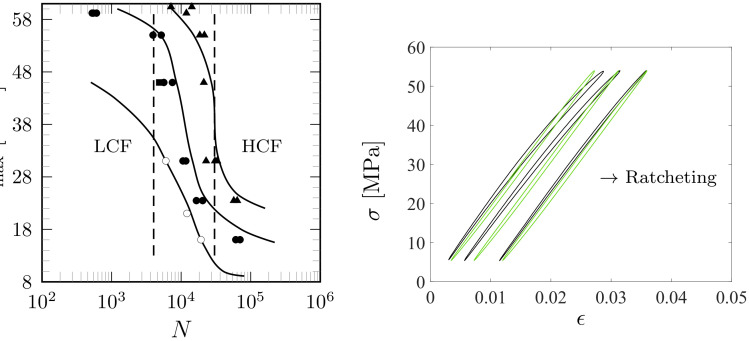
Predicted uniaxial S-N curves and data: f=5 Hz and R=−1 (the marker ∘ for data of flat specimen), R=0.1 (• flat specimen; *■* tubular specimen), and R=0.5 (*▲* flat specimen) (**left**). The 40th, 400th, and 3100th (prior to rupture) loops for the LCF region when R=0.1 (**right**). The green and black colors denote the model results and data, respectively.

**Figure 15:**
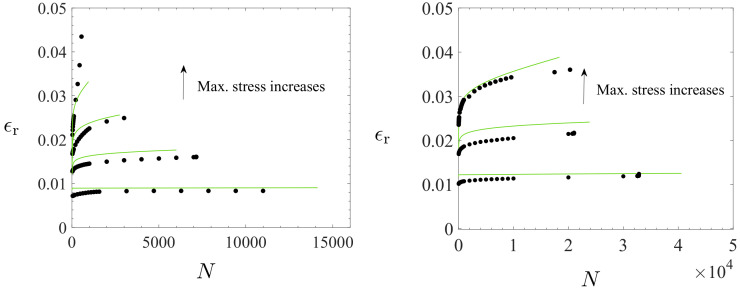
Ratcheting strains $\epsilon_{r}:=\frac{1}{2}\left(\epsilon_{\min}+\epsilon_{\max}\right)$ ($\epsilon_{\min}$ and $\epsilon_{\max}$ are the minimum and maximum strains in each cycle) when R=0.1 (**left**); the markers • and the green curves mean data and predicted results. The maximum stresses were 50%, 75%, 90%, and 97% of the ultimate tensile stress, 60 MPa. Ratcheting strains for R=0.5 (**right**). The maximum stress values were 50%, 75%, and 90%.

The value κ∼100…200, which defines the transition from the viscoelastic behavior (HCF region) to the plastic (LCF region), is limited and represents, therefore, preferably viscoplastic rather than viscoelastic (infinitely large value of κ) material behavior. When κ increases infinitely, damage evolution (13) reduces to

(49) $$dD=\frac{K}{1-BD}\exp\left(L\beta \right)d\beta \geq 0,$$

which a bulk form represents a long-term damage evolution similar to that of elastic metals [28]. However, the value κ for the transition is notable and is in accordance with the experimental observations and proposed constitutive theory for the irreversible, dissipative, and prolonged effects (area of the loops, cyclic creep, or ratcheting); the S-N curves show a reduced negative slope at a low number of cycles, a fairly straight slanting portion with an increased negative slope during long numbers of cycles (predicted essentially by κ), a re-reduced negative slope at a high number of cycles, and almost horizontal asymptote in the ultimate of the HCF region, cf. Figure 14(left). Also, the stress vs. strain loops and ratcheting strain responses for asymmetric loadings are well predicted, as shown in Figure 14(right) and Figure 15. The largest difference between the model predictions and data for fatigue life occurs in the ultimate of the HCF region when R=0.1, Figure 14(left). What is notable is that the influence of the stress amplitude is dominant [23]: the maximum stress for R=−1 remains well below compared to those for R>0 (up to 60 MPa).

Based on the curvatures of the observed S-N curves shown in Figure 14(left), it can be suggested that the LCF region covers less than 4000…10,000 cycles and the HCF region greater than 30,000…60,000 cycles; the S-N curves show a small negative slope at low number of cycles (LCF region) and a re-reduced negative slope or almost horizontal asymptote in the ultimate of the HCF region. These characteristics make the modeling challenging, and they are achieved by the proposed damage model (13), which is the composition of the LCF and HCF regions, including the curvature κ that completes the damage definition of how rapidly the transition between the asymptotes of the LCF and HCF regions is reached.

### 4.3. Microstructural Aspects

**Figure 16:**
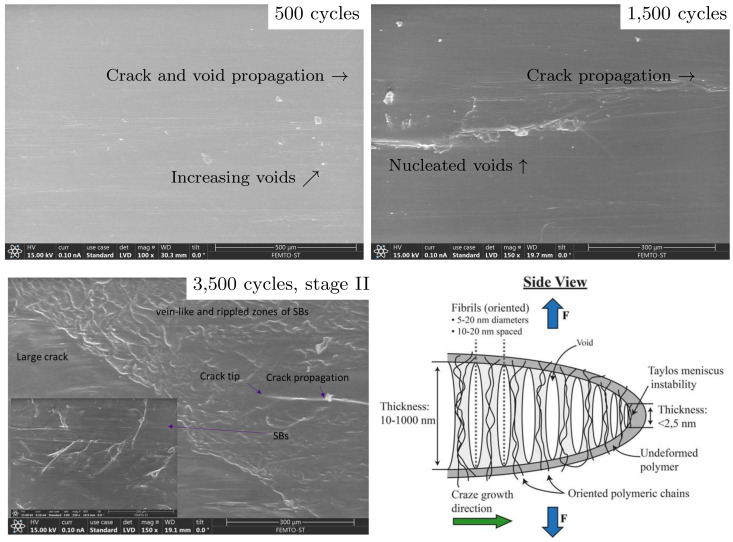
Microstructure at 500 cycles, 1500 cycles, and 3500 cycles (local SBs are shown in the inset; the tubular specimen used broke at 4800 cycles) when R=0.1 and $\sigma_{\max}=45$ MPa (75% of the ultimate tensile strength). Demonstration of the failure mechanism in the crack tip (bottom right).

**Figure 17:**
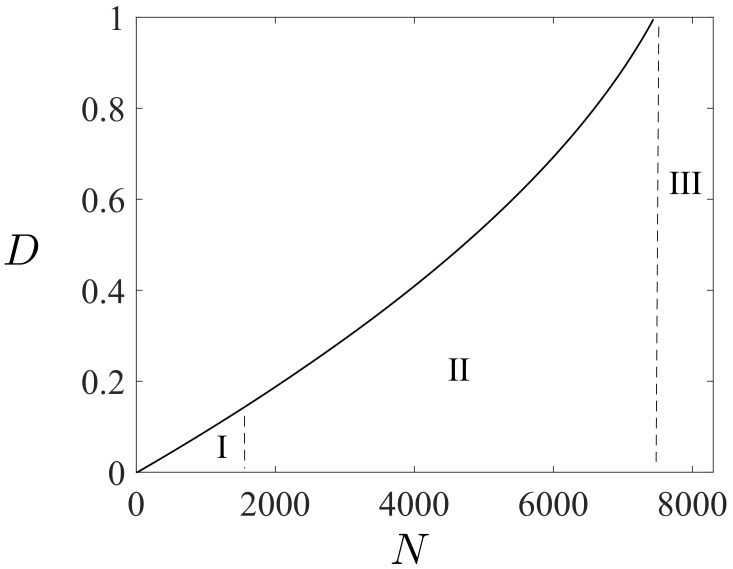
Predicted fatigue damage including the stages I–II when R=0.1, $\sigma_{\max}=45$ MPa, and f=5 Hz.

From the microstructural point of view, fatigue failure is due to the nucleation, growth, and coalescence of nanoscopic voids on the polymer chain network [47] governing over 90% of the entire lifetime [9,23,33,34,70]. In more detail, the increase in the free volume (representing a reduced chain density from the nano-to microscale [21,22,71]) has frequently been connected to the nucleation, growth, and coalescence of tiny voids affecting stable growth of shear bands (SBs) and microcracks (primary stage I) to form of macrocracks (secondary stage II) [20,47] and has been used to model damage [72], see Figure 16(top). The effect of the free volume φ is included in fatigue through the constitutive model as demonstrated in Figure 3, [7]. Furthermore, crazing, i.e., changes in the chain disentanglement and fibril (extended chain crystals or bundles between voids), explains the origin of plastic deformation for fatigue failure in crack tips at the stages I–II [13,44,45,73], see Figure 16(bottom right). Once most of the fatigue life (∼60%) is reached, vein-like and rippled zones of SBs start to develop, preventing the enlarging of the microcracks [74,75], see Figure 16(bottom left). This microstructural characteristic explains the long-term stable deformation behavior (and the large value of κ representing the long-term transition from LCF region to HCF) before complete failure (rupture) when the unstable macro-crack propagation progresses rapidly (tertiary stage III). The fatigue failure stages I–III and predicted fatigue damage representing the stages I and II are shown in Figure 17.

## 5. Conclusions

The article proposes a compact viscoelastic–plastic constitutive model and fatigue model for polymers, able to predict the macroscopic short- to long-term cyclic deformation behavior and fatigue life. The applied concept of a fatigue damage evolution and a moving endurance surface in the stress space averts the need for equivocal cycle-counting techniques. Meanwhile, the concept governs the effect of loading histories. Based on the proposed endurance function, a rule, as an alternative to the celebrated Gerber’s rule (1874) to quantify the Haigh diagram, was proposed, capable of considering the asymmetry of polymers between compression and tension. Furthermore, the similar macroscopic fatigue characteristics between polymers and metals motivated the use of the Basquin (HCF) and Coffin–Manson (LCF) formulas also for polymers; the fatigue parameters were determined in terms of the univocal, well-defined Basquin and Coffin–Manson parameters. Considering further research, the promising results motivate us to define and collect the Basquin and Coffin–Manson parameters for various polymers similar one has collected to metals. Due to the compact formulation and calibration procedure, the proposed model is easy to implement and use as a built-in feature in finite-element packages to perform in-depth looks at different polymer systems and loading scenarios. Therefore, model predictions can intensify the material development, and one can build digital (numerical) twins (a test setup and an up-to-date model representation of a test setup [76]) to avoid costly and time-consuming (trial-and-error) iterations needed for testing arrangements, as well as for model calibration. The digital twins can be used to predict the future mechanical behavior of the asset and to refine the control (manufacturing) and material development and characterization. Lastly, the integration of microstructural mechanisms with the proposed continuum damage model provides an interesting avenue for future research.

## Figures and Tables

**Table 1:** Elastic and viscoelastic model parameters for the PC (Lexan 223R).

Parameter	*E*	ν	${\dot{v}}_{0}$	α	$m_{1}$	$s^{\left(2\right)}$	$c_{1}\cdot 10^{-6}$	$\mu_{1,\mathrm{sat}}$	$\mu_{1}^{0}$
Unit	MPa		$s^{-1}$			MPa	MPa	MPa	MPa
Value	2000	0.37	0.031	0.204	0.19	12	4.5	2500	8000

**Table 2:** Viscoplastic parameters for the PC (φ(0)=0).

Parameter	$s_{0}$	$m_{0}$	$C_{R}$	*N*	$h_{0}$	$\hat{b}$	$g_{0}$	$s_{\mathrm{cv}}$	$\varphi_{\mathrm{cv}}$
Unit	MPa		MPa		MPa			MPa	
Value	28.0	0.037	14.0	1.65	3500	600	0.015	26.5	0.0013

**Table 3:** Fatigue model parameters for the PC applied. The final values b=0.18, $\sigma_{f}^{{}^{\prime }}=57$ MPa, $\xi_{f}^{{}^{\prime }}=0.7$, c=0.59, $\sigma_{c}^{{}^{\prime }}=53$ MPa, and $n_{c}=0.052$ were used. The strengths were $\sigma_{\mathrm{yi}}=40$ MPa and $\sigma_{u}=60$ MPa.

Source	$\sigma_{0}$ [MPa]	*a*	$a_{2}$ [MPa]^−1^	*C*	*K*	*L*	κ	$\tilde{L}$	*B*
Parameter	8.3	0.18	0.014	0.75	4.0$\cdot 10^{-5}$	1.1	160	18	0.7

## Data Availability

Data are contained within the article. We provide the source data underlying Figure 14 and Figure 15.

## Nomenclature

ϵ	uniaxial strain
***F***	deformation gradient
σ,s	stress and its deviatoric component
S−N	true stress range vs. number of cycles *N* to failure
$\sigma_{y}\;$	peak yield stress
$\sigma_{u}\;$	ultimate tensile strength
$\sigma_{a}$	stress amplitude
$\sigma_{m}$	mean stress
$\beta (\bar{\sigma },g;\sigma_{0})\;$	endurance function
$\bar{\sigma }\left(s,\alpha \right)\;$	equivalent stress
α(C)	fatigue variable (kinematic)
C	model (material) parameter
$\sigma_{0},\;\sigma_{+0}\;$	fatigue strengths for alternating and pulsating uniaxial loads
$g(I_{1};a,a_{2})\;$	function in β for the mean stress $\sigma_{m}$
$I_{1}\;$	first stress invariant
$a,\;a_{2}\;$	slope parameters of the Haigh diagram for low and high values of $\sigma_{m}$
*D*	fatigue damage
HCF, LCF	high-cycle fatigue, low-cycle fatigue
K,L,C	fatigue damage parameters for HCF
$\tilde{L},\;\kappa \;$	fatigue damage parameters for LCF
R	stress ratio $\sigma_{min}/\sigma_{max}$
ζ	stress ratio $\sigma_{a}/\sigma_{0}$
$\sigma_{f}^{{}^{\prime }},\;b\;$	parameters of Basquin equation
$\xi_{f}^{{}^{\prime }},\;c\;$	parameters of Coffin-Manson formula
$\sigma_{c}^{{}^{\prime }},\;n_{c}\;$	parameters of Ramberg-Osgood formula
$\epsilon_{r}\;$	ratcheting strain
SBs	Shear bands

## Appendix A. Integration of the Fatigue Model

An implicit Euler scheme was used for updating fatigue model variables (9) and (13). Their integration resulted in the following nonlinear residual functions:

(A1) $$\begin{array}{cc} R & =\alpha -\alpha_{n}-d\alpha ,\\ R & =D-D_{n}-dD, \end{array}$$

where n refers to the last state of equilibrium, and the increment of the endurance function (2) needed in them is

(A2) $$d\beta =\frac{1}{\sigma_{0}+C\bar{\sigma }}\left(\frac{3}{2}\frac{(s-\alpha ):ds}{\bar{\sigma }}+\left(a+2a_{2}I_{1}\right)\mathrm{trace}\left(d\sigma \right)\right)$$

In (A1), $dD=\dot{D}dt$, $d\alpha =\dot{\alpha }dt$, and dt is the time increment. In the numerical calculations, dt is replaced by the time step Δt. Since the calculation of long fatigue lives is time-consuming, it is worth investigating how to reduce the time step. Considering the applied sinusoidal cyclic loads with the stress amplitude $\sigma_{a}$ and frequency 1 Hz. An error in the area of one cycle is

$$e=\sigma_{a}\left(\int_{0}^{1}\sin\left(2\pi t\right)dt-\sum_{i}^{I}\left(\Delta t\sin\left(2\pi n\Delta t-\frac{\Delta t}{2}\right)\right)\right)=\sigma_{a}\left(\frac{1}{\pi }-\sum_{i}^{I}\left(\Delta t\sin\left(2\pi n\Delta t\right)\right)\right),$$

where 2πnΔt>>Δt/2 was taken into consideration and I=1/Δt. Requiring the error always is less than 1%, one obtains for $\sigma_{a}=9$ MPa (R=−1) Δt∼0.06 s, $\sigma_{a}=40$ MPa (R=−1) Δt∼0.004 s, and for $\sigma_{a}=6$ MPa (R=0.5) Δt∼0.015 s. Then, the fitted time step can be approximated by

$$\Delta t=c_{1}{\left(\frac{\sigma_{0}}{\sigma_{a}}\right)}^{c_{2}}\exp\left(-c_{3}\frac{\sigma_{m}}{\sigma_{a}}\right),$$

where $\sigma_{a}>0$ holds for fatigue loads and $c_{1}=0.065$, $c_{2}=1.7$, and $c_{3}=0.7$ are fitted parameters.
